# Designing an optimized strategy for extracellular expression of recombinant human TNF-α in *Escherichia coli*

**DOI:** 10.3389/fbioe.2026.1897529

**Published:** 2026-08-28

**Authors:** Akanksha, Sounak Dasgupta, Priyanka Jain, Manisha Kuanr, Gaurav Pandey, Krishna Jyoti Mukherjee

**Affiliations:** 1 University School of Biotechnology, Guru Gobind Singh Indraprastha University, Dwarka, New Delhi, India; 2 Department of Biochemical Engineering and Biotechnology, Indian Institute of Technology Delhi, Hauz Khas, New Delhi, India

**Keywords:** *E. coli*, extracellular export, human TNF-α, knockouts, SecA/SecB

## Abstract

Extracellular protein expression in *Escherichia coli* is an elegant solution that addresses the complex issue of protein misfolding while simultaneously simplifying downstream processing steps. Human TNF-α was chosen as the target protein for export since it is a therapeutically important cytokine. Different genomic knockouts were tested for the ability to sustain and enhance protein expression, and BW25113 Δ(*elaA* + *cysW*) knockout was found to give a sustained and high level of expression. To improve secretion, various tags were tested, and the MBP tag at the N-terminal end was found to give maximum enhancement in the export of hTNF-α. Even the linker peptide was found to play a critical role in export, with the Ek linker giving the highest extracellular secretion, while the intein sequence completely blocked export. The co-expression of pSecAB, which is involved in protein transport to the periplasm, was also found to be helpful in enhancing extracellular protein titers. Interestingly, pelB performed poorly as compared to the native signal sequence of MBP, which gave better results. Culture conditions were optimized, and it was observed that growing cells in TB medium at a temperature of 25 °C, coupled with a pulse of concentrated nutrients at 24 h, led to a very high extracellular accumulation of ∼1.3 g/L of MBP-hTNF-α in shake flask culture. The protein was purified and tested using L929 cells for bioactivity. Thus, a combination of genomic and bioprocess strategies allowed us to obtain high levels of soluble and active extracellular expression of hTNF-α, making this a very attractive strategy for protein production.

## 1 Introduction

Extracellular export resolves many of the problems that plague the *Escherichia coli* expression system, where the protein is typically retained in the cytoplasm. The biggest of these is protein misfolding, which occurs due to the crowded molecular environment in the cell. Multiple solutions like co-expression of disulfide isomerase, chaperones, and slowing down the translation rates have been explored with reasonable success (de Marco et al., 2005; Gąciarz et al., 2017; Gopal and Kumar, 2013). Secretion to the periplasm is considered a superior strategy for obtaining properly folded proteins, given that its oxidative environment promotes proper disulfide bond formation (de Marco, 2009; Manta et al., 2019). Export from periplasm to the supernatant is even better because intermolecular interactions between proteins are rendered insignificant, promoting proper folding. The other obvious advantage is that subsequent downstream processing steps are simplified, and the absence of cellular contaminants like host cell proteins, DNA, RNA, and endotoxins improves both recovery and product quality, especially when making therapeutic proteins. Since *E. coli* remains the host of choice for the production of recombinant proteins, multiple attempts have been made to efficiently export proteins to the extracellular medium.

One successful attempt at export was to knock out the *lpp* gene encoding Braun’s lipoprotein, which anchors the peptidoglycan to the outer membrane and maintains structural stiffness and integrity. The *lpp* knockout strain showed higher membrane permeability than the wild-type strain and has been successfully utilised for the secretion of various recombinant proteins in the extracellular medium (Shin and Chen, 2008; Ding et al., 2019). However, multiple proteins leach out in the extracellular medium, compromising product quality. Similarly, addition of chemical additives such as glycine, Triton X-100, as well as deletion of the *pal*, *mrcA*, *mrcB, dacA, dacB, and dacC* genes, also perturb membrane permeability and make the cell leaky (Kleiner-Grote et al., 2018; Chen et al., 2014; Yang et al., 2018; Hu et al., 2019). Thus, host cell protein contamination is a serious challenge while using these strategies. Cell wall-deficient L-form cells were also created to support extracellular secretion. Low secretory yields, sensitivity to environmental changes, and slow growth made them unfit for further industrial applications (Gumpert and Hoischen, 1998; Lokireddy et al., 2025). Co-expression of *kil* genes has been used to export hGH, hTNF-α, rhIgG-Fc, hIL-2, streptavidin, and β-glucanase in the extracellular medium. However, high expression of *kil* genes is considered to be lethal for the producing strain therefore, careful optimization is necessary (Wal et al., 1995; Sommer et al., 2010; de Marco, 2009). Apart from increasing the bacterial leakiness, naturally secreted protein fusion partners such as yebF have also been explored (Zhang et al., 2005; Fisher et al., 2011).

The extracellular yields of multiple disulfide-linked proteins reported till now are extremely low. Also, the above-mentioned export strategies essentially rely on perturbing outer membrane permeability, thus compromising the key benefit of extracellular export, i.e., low host cell protein contamination. This is why designing an extracellular export strategy remains a challenge, which requires a paradigm shift in our approach to this problem. The first step in this design is to note that extracellular export is a slow process, and significant accumulation in the extracellular medium can only take place when recombinant protein expression is sustained for long time periods. We had previously designed knockout strains that block the cellular stress response (CSR), which is responsible for feedback inhibition of both growth and protein expression within a few hours of induction (Sharma et al., 2020; Guleria et al., 2020). In this work, we leveraged these knockouts, which are known to sustain expression for very long periods, and combined them with genetic as well as bioprocess strategies to improve extracellular export.

Initially, we chose the best performer, a double knockout (DKO) BW25113Δ(*elaA + cysW*), among all the knockouts created in our previous study. Human TNF-α was chosen as the model protein for export because it is a therapeutically important cytokine involved in inflammation, apoptosis, and immune system development. Expression of hTNF-α as a soluble recombinant protein in *E. coli* requires multiple optimization steps, and the yields obtained are fairly low (Castiñeiras et al., 2018; Papaneophytou and Kontopidis, 2012; Hoffmann et al., 2010).

Multiple targets were chosen to enhance extracellular export. The first of these was vector design involving the selection of signal peptides, fusion tags, and linker peptides to the protein of interest. The second was to optimize cultivation conditions that support maximum extracellular secretion at the shake-flask level. The third was to determine the effect of co-expressing genes related to export. The high-level of soluble and bioactive hTNF-α obtained by combining these strategies makes this a very attractive strategy for protein production.

## 2 Materials and methods

### 2.1 Strains and plasmids

All the plasmids used in this study were constructed and sequenced previously in the lab. *Escherichia coli* BW25113 was obtained from the Keio collection and used as a control. The double knockout (DKO) *E. coli* strain Δ(*elaA + cysW*), used for expression studies, was constructed by Sharma et al., 2020 using BW25113 as the parent strain. No other knockouts were made in the DKO strain.

### 2.2 Shake flask expression studies

DKO was transformed with various vectors as listed in Table 1. A single colony of each was inoculated in 5 mL TB medium as the primary inoculum. From the overnight-grown primary inoculum, 1% of the culture was inoculated in 25 mL TB+0.4% glycerol medium and incubated at 25 °C. Once the culture reached an OD_600_ of 1.0–1.5, induction was done using 0.5 mM IPTG. Following induction, the cultures were incubated further at 25 °C for 20 h post-induction. Samples were collected at 20 h post-induction to analyze the export profile. For expression analysis, the culture was first diluted to 1 OD_600_, and 1 mL of this diluted culture was pelleted. The pellet was resuspended in 100 µL of 1X reducing dye, lysed by boiling for 5 min, and used for loading on SDS-PAGE as a whole cell lysate sample. Supernatant samples were prepared by directly using 80 µL of culture supernatant, to which 20 µL of 5X reducing dye was added. Different volumes of this were loaded on SDS-PAGE depending on the concentration of the target protein. All the experiments were done in triplicate, and the SDS-PAGE samples were prepared similarly for further optimizations.

**TABLE 1:** Shows combinations of signal peptides and protein fusion partners used for the extracellular export of hTNF-α in DKO.

Plasmid name	Signal peptide	Carrier protein	Antibiotic marker (working conc.)	Molecular weight of fusion protein
pET22bpelB-hTNF-α	pelB	—	Ampicillin (100 μg/mL)	∼19.7 kDa
pBAD24pelB-PA-hTNF-α	pelB	SpA	Chloramphenicol (35 μg/mL)	∼46 kDa
pBAD24pelB-Ans-hTNF-α	pelB	First 169 amino acids of Asparaginase	Chloramphenicol (35 μg/mL)	∼37 kDa
pBAD24yebF-Ek-hTNF-α	pelB	YebF	Chloramphenicol (35 μg/mL)	∼31 kDa
pMAL-p2XMBP-hTNF-α	malE	—	Ampicillin (100 μg/mL)	∼63 kDa

Ek, Enterokinase cleavage site.

### 2.3 Pulse time and composition optimization

Growth studies of DKO carrying pMAL-p2XMBP-hTNF-α were conducted for uninduced and induced cultures till 45 h post-induction.

To optimize the pulsing composition, secondary culture of DKO carrying pMAL-p2XMBP-hTNF-α was grown at 25 °C with 100 μg/mL ampicillin. Induction was done at an OD_600_ of 1–1.5 using 0.5 mM IPTG. The culture was divided equally into 9 flasks after 20 h of induction, each carrying 25 mL of induced culture. A pulse of different composition was added to all flasks, viz. 1) No pulse, 2) 0.2% Glycerol, 3) 0.4% Glycerol, 4) 0.05% Tryptone and yeast extract (Y.E.) individually, 5) 0.1% Tryptone and Y.E. individually, 6) 0.2% Glycerol+0.05% Tryptone and Y.E. individually, 7) 0.4% Glycerol+0.05% Tryptone and Y.E. individually, 8) 0.2% Glycerol+0.1% Tryptone and Y.E. individually, 9) 0.4% Glycerol+0.1% Tryptone and Y.E. individually. No glycerol was added at a pH below 7.0 of the culture medium. Continuous production was ensured by adding 0.25 mM IPTG and 50 μg/mL ampicillin to prevent plasmid instability and to ensure that the plasmid-free cell population remains at negligible levels. Replica plating is not an accurate method to estimate instability because plasmid-containing cells often lose their colony-forming ability post-induction. This leads to overrepresentation of plasmid-free cells on the plate (Yazdani and Mukherjee, 2002). A better, though indirect strategy is to look for a sudden increase in the OD_600_ of the culture post-induction, which signals the emergence of plasmid-free cells. Since we had previously observed this sudden rise in OD_600_ 20 h post-induction, we added an antibiotic pulse, which completely prevented this rise. This demonstrates that the re-application of selection pressure is necessary to maintain plasmid-containing cells in the culture, and in our case, the OD_600_ of the culture did not increase after 20 h. Samples were drawn to check OD_600_ at different time points. Supernatant samples of 40 h post-induction were prepared and loaded on 12% SDS-PAGE to analyze the extracellular yields at different conditions tested.

### 2.4 Temperature optimization

Temperature optimization was carried out at two different temperatures, including 18 °C and 25 °C. A third condition was also tested where the flask from 18 °C was shifted to 25 °C post-induction 20 h. In all conditions, the cells were induced with 0.5 mM IPTG when the OD_600_ reached ∼1.5. All the culture flasks were pulsed with 0.4% glycerol+0.05% tryptone and 0.05% yeast extract post-induction 20 h. 20 and 40 h post-induction whole cell lysate and supernatant samples were prepared and run on 12% SDS-PAGE.

Protein-partitioning samples were prepared using a 5 OD_600_ equivalent cell pellet. First, the pellet was resuspended in Tris-Cl pH 8.0, and lysis was done by sonication for 10 min at 40% amplitude with 10 s on/off cycle. To separate soluble and insoluble fractions, centrifugation was done at 10,000 X ‘g’ for 30 min. 5X sample loading dye was added to the supernatant and the pellet. An equal amount of the protein was loaded on the 12% SDS-PAGE.

### 2.5 Cloning of the enterokinase cleavage site between MBP and hTNF-α

An enterokinase cleavage site was inserted between MBP and hTNF-α to obtain native hTNF-α for the biological activity assay. hTNF-α gene was amplified using EcoRI-Ek site (N-terminus) carrying forward primer (5′-ATA​CCG​GAA​TTC​GAT​GAC​GAT​GAC​AAA​GTT​CGT​AGT​AGT​AGT​CGT​ACC​C-3′) and HindIII (C-terminus) carrying reverse primer (5′-CGC​CAA​GCT​TTC​ACA​GCG​CGA​T-3′). Phusion polymerase was used for amplification, and the elongation was done for 30 s. The obtained PCR product was digested with both enzymes along with the empty pMAL-p2X vector. Digested products were then gel-eluted to set up the ligation reaction overnight at 4 °C. The ligation mixture was transformed into DH5α cells and plated on an ampicillin-containing agar plate. Next day, colony PCR was done to check for any positive clones using gene-specific forward and reverse primers. Further confirmation of positive clones was done by setting a double digestion reaction using EcoRI and HindIII restriction enzymes. Final confirmation of the construct was done by Sanger sequencing.

### 2.6 Co-expression of pMALp2XMBP-Ek-hTNF-α with p^PROLAR.A^SecAB in a shake flask

Confirmed clone of pMAL-p2XMBP-Ek-hTNF-α was co-transformed with p^PROLAR.A^SecAB in DKO. A single colony was inoculated in 5 mL of TB media supplemented with ampicillin (100 μg/mL) and kanamycin (50 μg/mL). The overnight-grown culture was used as the primary inoculum, and 1% of the primary inoculum was inoculated into the secondary culture media. 25 mL of TB+0.4% glycerol media with appropriate antibiotics was used to conduct expression studies. The secondary cultures were incubated at 25 °C with constant shaking at 200 rpm. Pulsing was done at 20 h post-induction using 0.4% Glycerol+0.05% Tryptone and 0.05% yeast extract. Samples were collected 20 and 40 h post-induction for comparative analyses.

### 2.7 Purification of MBP-Ek-hTNF-α using amylose affinity resins

Affinity purification was done using amylose resins at a high flow rate. First, the collected supernatant was diafiltered using a 30 kDa Hydrosart membrane to bring the protein into the MBP-binding buffer (20 mM Tris pH 8.0, 400 mM NaCl, 1 mM EDTA) for purification. Meanwhile, the resins were equilibrated with the binding buffer, and the sample was loaded with a flow rate of 4 mL/min. The binding was followed by washing of the resin using the same buffer to remove non-specific proteins. The target protein was eluted using 20 mM Tris pH 8.0, 200 mM NaCl, and 50 mM maltose at a 2 mL/min flow rate. The percentage recovery of the purified fusion protein was calculated by densitometry. The diafiltered load sample and elute fraction were first volume corrected and then compared with the original supernatant fraction by loading different amounts on the SDS-PAGE.

An overnight digestion reaction at 4 °C was set up using bovine enterokinase. The digested fraction was used for further polishing to obtain native and pure hTNF-α.

### 2.8 Polishing of hTNF-α by using DEAE resins

Following digestion, the reaction mix was first buffer-exchanged to 20 mM Tris pH 7.5, 50 mM NaCl by multiple centrifugation steps using 3 kDa centricon tubes. Weak anion-exchange DEAE resins were used for this step which bound the MBP, while the target protein was collected in the flow-through fraction.

### 2.9 Biological activity of purified recombinant hTNF-α (rhTNF-α)

The bioactivity of the purified recombinant hTNF-α was evaluated by cytotoxicity assay of L929 cells with modifications as described by Evans (2000). L929 cells were cultured in Minimum Essential Medium (MEM), 10% fetal bovine serum (FBS), and 1% penicillin-streptomycin for cytotoxicity measurement. The experiment was carried out in 96-well plates. Each well was filled with 100 µL of culture medium with 2 × 10^5^ cells/mL, and the plates were incubated overnight in a 37 °C/5% CO_2_ cell incubator. After 24 h of incubation, the culture medium was replaced with 100 μL of culture medium containing 1 μg/mL actinomycin D and recombinant human TNF-α (0.1–1,000 pg/mL), followed by incubation for 24 h. Subsequently, cells were stained with 10 μL of CCK-8 solution for 2 h, and the optical density was determined at 450 nm and 620 nm. Wells containing culture medium only and those containing untreated cells were set up as the blank control and normal control, respectively. Samples were tested in triplicate in three independent experiments.

## 3 Results

### 3.1 Screening of signal peptides and carrier proteins for enhancing the export of hTNF-α to the culture medium

**FIGURE 1:**
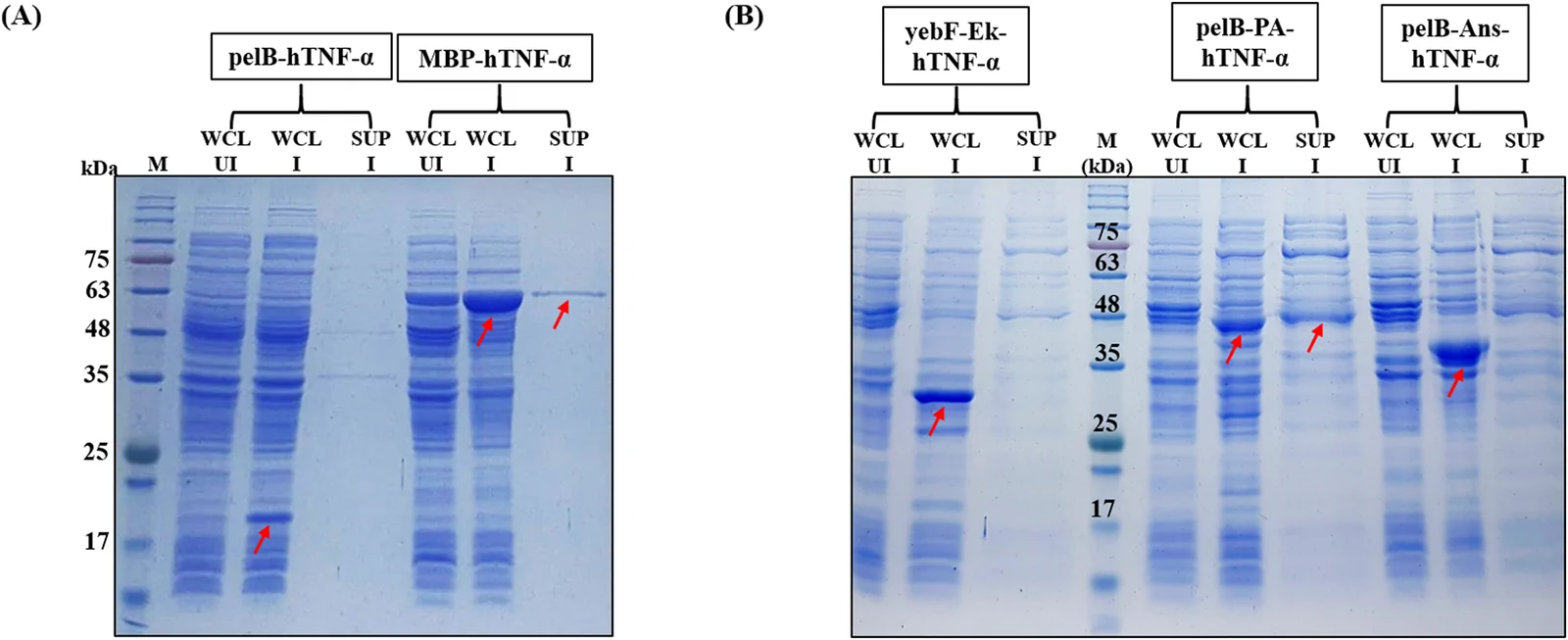
**(A,B)** SDS-PAGE showing localization of tagged hTNF-α expressed using DKO strain and BL21 (DE3) in the case of pET22bpelB-hTNF-α in 20 h post-induction samples. Export was only observed in the case of MBP-hTNF-α and pelB-PA-hTNF-α. M-molecular weight marker; WCL, 10 µL of 10 OD_600_ equivalent whole cell lysate sample; SUP, 20 µL of culture supernatant; UI, Uninduced; I, Induced.

A preliminary study to measure the relative levels of hTNF-α export was carried out by screening multiple signal peptides and naturally secreted proteins as fusion partners (Table 1). Intracellular accumulation was observed in all the clones at the appropriate size, given that they carried tags of different molecular weights (Figures 1A,B). However, among all the tested combinations, only MBP and pelB-PA showed extracellular protein export. Moreover, true export was only observed when MBP was used as a fusion protein along with its native signal peptide. Though the level of secretion was low, the fusion protein in the extracellular medium was highly pure. On the other hand, target protein-specific export was not observed with pelB-PA. The supernatant fraction contained multiple bands representing leakage of non-specific host cell proteins. Therefore, the MBP-tagged hTNF-α was chosen for further optimization to improve the extracellular yields.

### 3.2 Optimization of pulsing time

**FIGURE 2:**
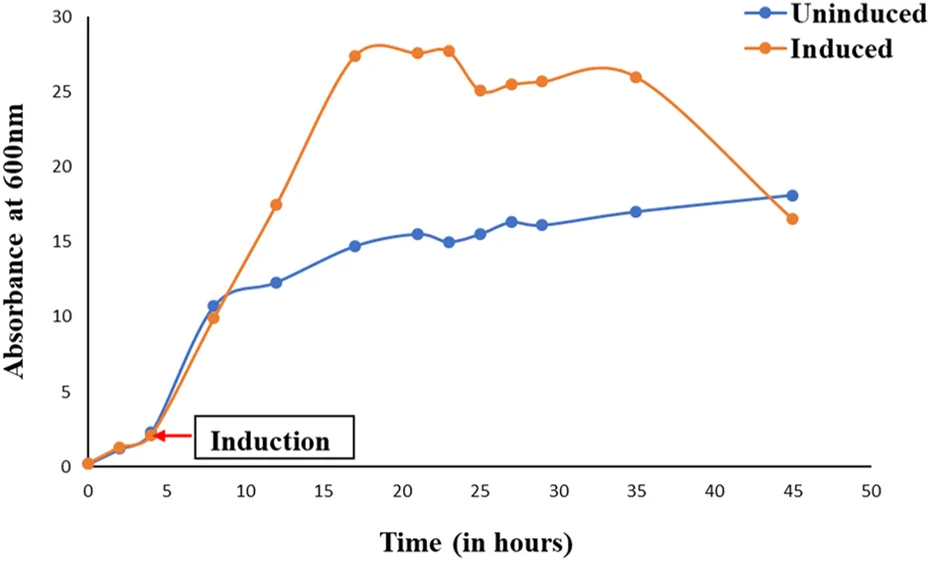
Graph showing growth profile of DKO expressing MBP-hTNF-α in uninduced and induced cultures. OD_600_ declines slightly after approximately 20 h of induction.

Given the fact that intracellular accumulation was quite high, we first considered incubating the cultures for a longer period. Since this could result in nutrient depletion and poor cellular health, the addition of a pulse of nutrients was adopted to prevent lysis. To determine the best time point for pulse addition, the growth profile of the induced and uninduced cells was monitored. A slight decline in OD_600_ of the induced culture was observed after 20 h post-induction (Figure 2). We therefore decided to add the pulse at this time point, i.e. 20 h post-induction. Considering that the pulse addition would keep the cells viable for an extended period, we decided to provide a longer time for the buildup of extracellular titers and harvest the cells 40 h post-induction.

### 3.3 Optimization of pulsing composition

**FIGURE 3:**
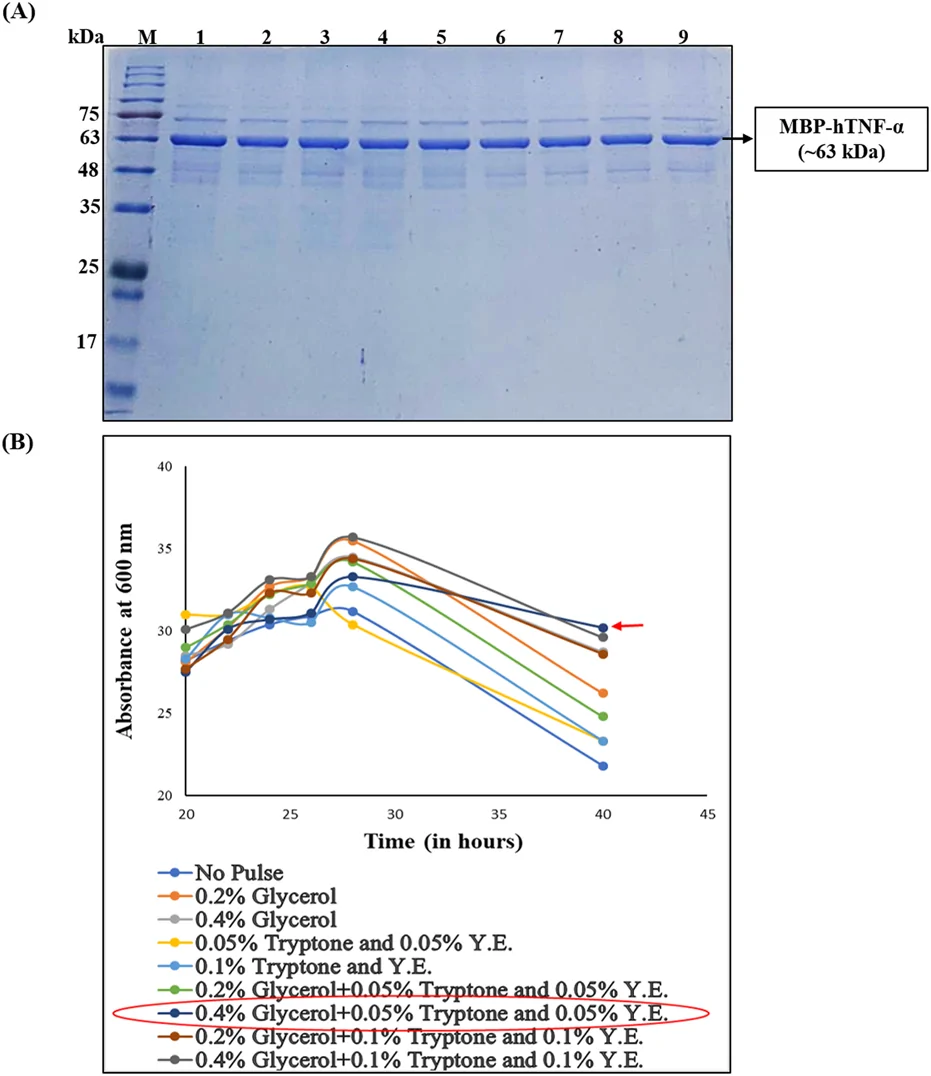
**(A)** SDS-PAGE showing 5 µL extracellular supernatant sample of MBP-hTNF-α expressed till 40 h post-induction using the DKO strain. Different compositions of pulses were added after 20 h of induction to support better growth. **(B)** Growth curve showing effects of different pulse compositions on induced cultures of DKO strain expressing MBP-hTNF-α. Red arrow and red circle showing the best optimized condition for further studies. Lane M-Molecular weight marker; Lane 1-No pulse; Lane 2%–0.2% glycerol; Lane 3%–0.4% glycerol; Lane 4%–0.05% tryptone and 0.05% yeast extract.; Lane 5%–0.1% tryptone and 0.1% yeast extract; Lane 6%–0.2% glycerol+0.05% tryptone and 0.05% yeast extract; Lane 7%–0.4% glycerol+0.05% tryptone and 0.05% yeast extract; Lane 8%–0.2% glycerol+0.1% tryptone and 0.1% yeast extract; Lane 9%–0.4% glycerol+0.1% tryptone and 0.1% yeast extract.

A varying concentration of glycerol (as a carbon source) and tryptone and yeast extract (as a complex nitrogen source) was tested in different combinations since an imbalance in the C/N ratio can decrease product yield. High glycerol can lead to a reduction in pH due to acetate buildup, which inhibits product formation, whereas low glycerol can reduce cell viability. Therefore, multiple combinations of C/N ratios were added, and fairly high levels of extracellular export of MBP-hTNF-α were observed under all the conditions tested (Figure 3A). However, the growth profile showed that 0.4% glycerol+0.05% tryptone and 0.05% yeast extract was an optimal pulse composition to support the long-term growth of DKO cells expressing MBP-hTNF-α (Figure 3B). The pH across all the conditions tested was observed as ∼7.0, which, after an initial fall, rose continuously to about 8.0. When the pulse of nutrients was added, again after a slight fall in pH, it rose slowly to 8.5. This rise in pH was critical because this high pH helped in improving the export of the protein.

### 3.4 Effect of incubation temperature on export of MBP-hTNF-α

Membrane permeability and protein solubility are important factors when targeting extracellular export of recombinant proteins. Low incubation temperatures promote solubility, whereas high temperatures increase both expression rates and permeability of the cell membrane, leading to better export. Low temperatures reduce translation rates and prevents overwhelming the translocation system, while fast translation rates at high temperatures generally result in the accumulation of misfolded aggregates. Therefore, to understand the effect of cultivation temperature on the export of MBP-hTNF-α, 18 °C and 25 °C were selected for optimization. Another growth strategy where the cultivation temperature was shifted from 18 °C to 25 °C after 24 h was also tested. The idea here was to increase solubility by initially incubating at low temperatures and then shifting to a higher temperature for efficient export.

**FIGURE 4:**
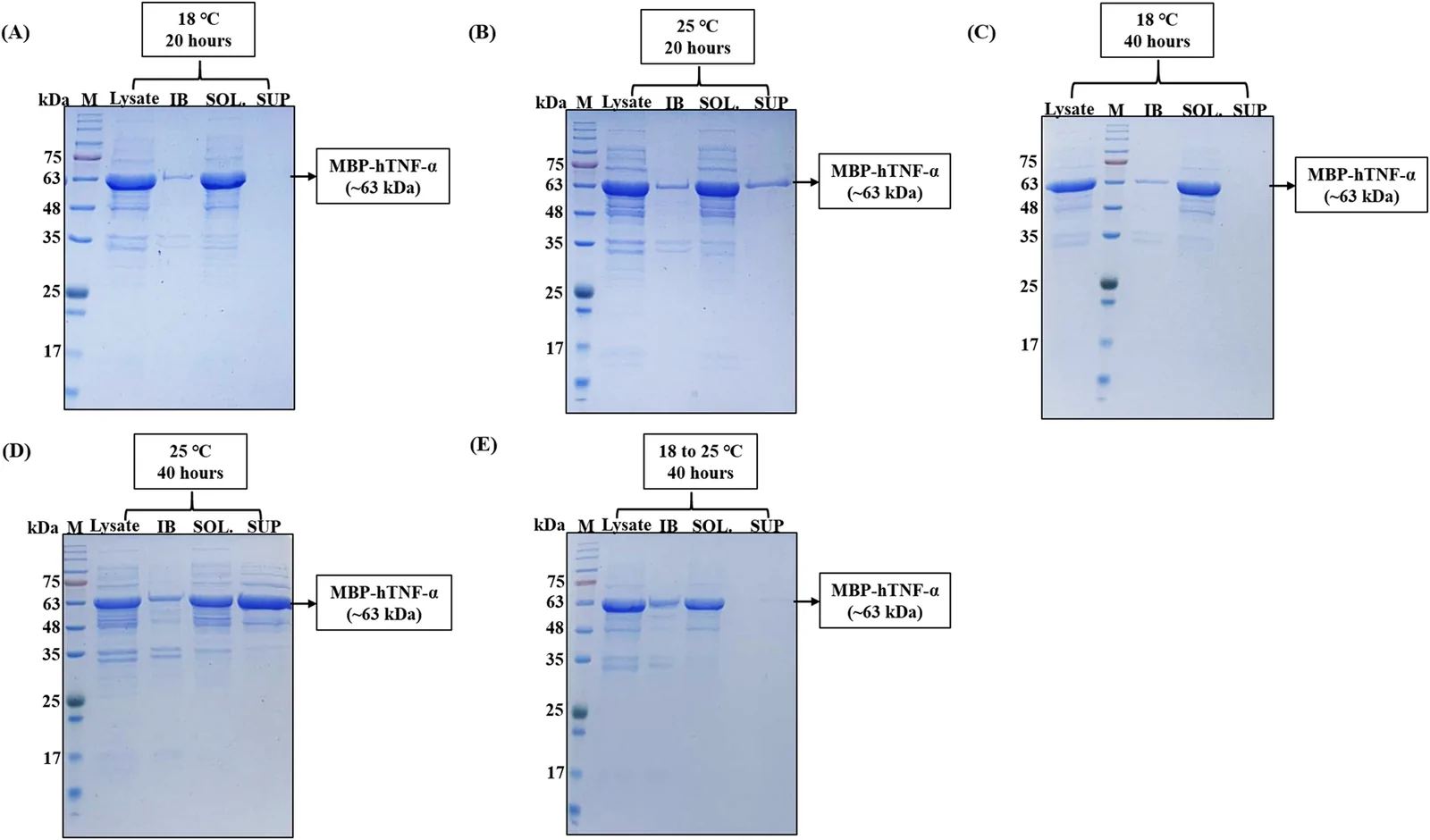
SDS-PAGE showing partitioning and localization of MBP-hTNF-α in post-induction **(A)** 20 h samples of culture incubated at 18 °C **(B)** 20 h samples of culture incubated at 25 °C **(C)** 40 h samples of culture incubated at 18 °C **(D)** 40 h samples of culture incubated at 25 °C **(E)** 40 h samples of culture shifted from 18 °C to 25 °C. Higher export of fusion protein was observed at 25 °C as compared to low incubation temperature (18 °C). M, Molecular weight marker; Lysate, Culture before sonication; IB, Insoluble fraction; SOL., Soluble fraction; SUP, 20 µL of culture supernatant.

We observed soluble production of MBP-hTNF-α at both temperatures due to the presence of the N-terminal MBP tag. However, a drastic drop in extracellular titers was observed when the temperature was lowered to 18 °C (Figures 4A–D). Extracellular yields did not improve even after shifting the flask to 25 °C from 18 °C after 24 h of incubation, where a very faint band of MBP-hTNF-α was observed in the supernatant fraction (Figure 4E). Since the protein remained soluble even when the cultivation temperature was kept at 25 °C throughout, it was only logical to keep this as the optimal temperature for the export of recombinant fusion protein.

### 3.5 Co-expression of p^PROLAR.A^SecAB

**FIGURE 5:**
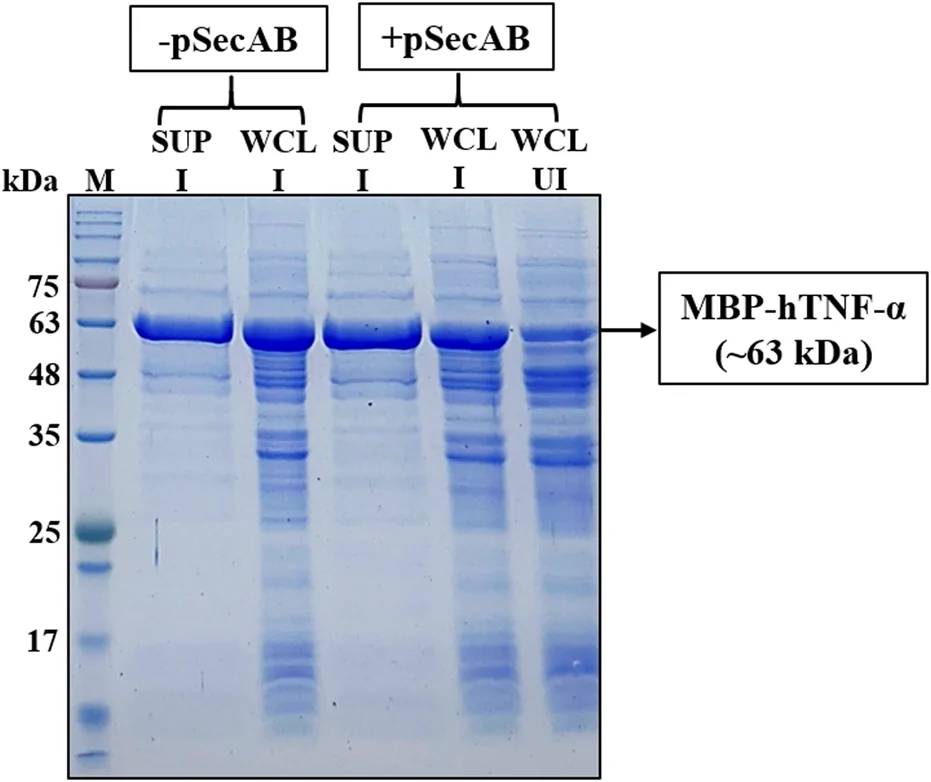
SDS-PAGE showing co-expression of MBP-hTNF-α and p^PROLAR.A^SecAB in DKO strain till post-induction 40-h. Co-expression of SecA and SecB improved export of MBP-hTNF-α by ∼1.3 fold. M, Molecular weight marker; UI, Uninduced; I, Induced; WCL, 10 µL of 10 OD_600_ equivalent whole cell lysate sample; SUP, 20 µL of culture supernatant.

Low extracellular yields with the MBP-tagged construct could be due to the slow diffusion rates of protein to the extracellular medium as a consequence of inefficient translocation to the periplasm. Previous transcriptomic analysis of cultures producing recombinant proteins has shown a downregulation of multiple genes post-induction, including those involved in translocation (Guleria et al., 2020). Therefore, co-expression of critical components can help in improving export. SecA and SecB are crucial components of the Sec-dependent translocation system. While SecB binds to the nascent polypeptide and keeps it in a translocation-competent state, SecA recognizes SecB and acts as a receptor. SecA also initiates protein translocation by triggering structural changes in SecYEG (Mergulhão et al., 2005; Lycklama a Nijeholt and Driessen, 2012). We therefore co-expressed SecA and SecB to test the hypothesis that secretion to the periplasm is a bottleneck to export. We observed a 1.3-fold improvement in the export of MBP-TNF-α (Figure 5) when SecA and SecB were co-expressed in comparison to the control. We also checked for the fold improvement in export yields of MBP-hTNF-α by replacing the malE (native signal sequence of MBP) with pelB. However, no significant difference was observed with the export yields actually being slightly lower when pelB was used as a signal sequence (Supplementary Figure S1).

### 3.6 BW25113 vs. DKO

To demonstrate that the sustained expression capability of the DKO was a necessary condition for high extracellular titers, a comparative analysis of export yields between BW25113 and DKO was conducted under optimized conditions.

**FIGURE 6:**
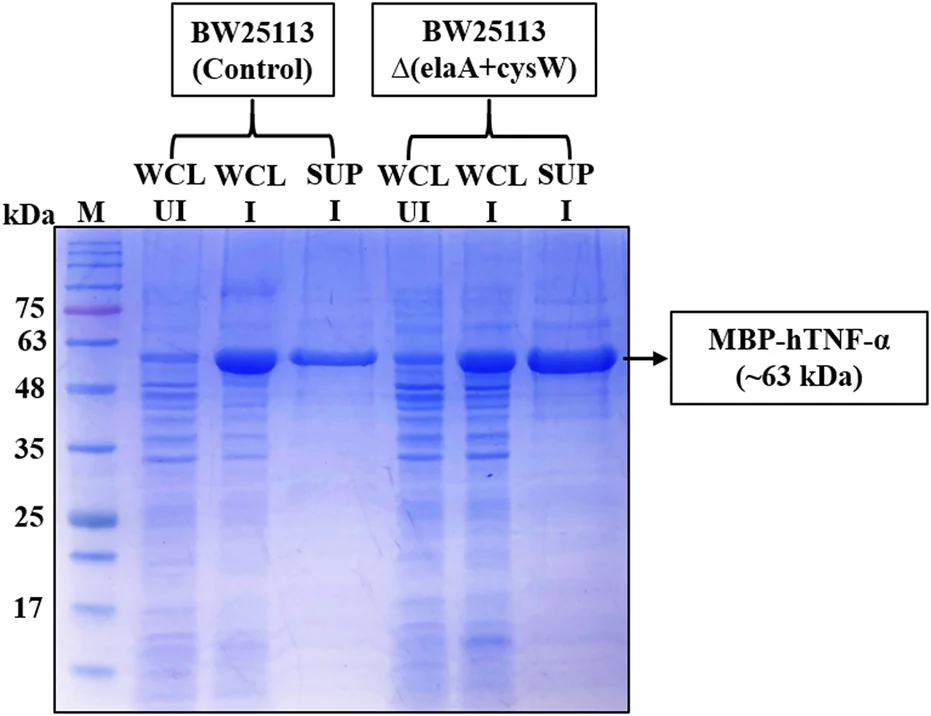
SDS-PAGE showing the co-expression profile of MBP-hTNF-α and p^PROLAR.A^SecAB in BW25113 and DKO *Escherichia coli* strains. Expression studies were conducted till post-induction.40 h. DKO showed high extracellular export of MBP-hTNF-α as compared to the wild-type control strain. M, Molecular weight marker; UI, Uninduced; I, Induced; WCL, 10 µL of 10 OD_600_ equivalent whole cell lysate sample; SUP, 20 µL of culture supernatant.

As seen in Figure 6, the intracellular accumulation of MBP-TNF-α in both strains was similar. However, a large ∼2.3-fold increase in export of MBP-hTNF-α was observed in the DKO strain compared to control *E. coli* BW25113. This demonstrates that DKO is a superior strain because of its ability to counter the cellular stress response and hence sustain expression for longer periods of time, an essential prerequisite for enhancing extracellular titers. The optimized conditions were also tested using a standard protein expression strain, i.e., BL21 (DE3), which showed much lower intracellular as well as extracellular expression of MBP-hTNF-α (Supplementary Figure S4).

### 3.7 Intein vs. enterokinase as a cleavage site

Removal of tags from the target protein is necessary for achieving its native Conformation and biological activity. Fusion tags could elicit an immune response and are thus undesirable for commercial preparations of therapeutic proteins or for further studies (Feng et al., 2014). To simplify this removal, we decided to use inteins, which are self-cleavable affinity tags, and the target protein could be separated easily from fusion tags by modulation in pH, temperature, or ionic strength. On the other hand, enterokinase was chosen as a second linker since it specifically recognizes DDDDK↓ as a cleavage site and does not leave any extra amino acid, thus faithfully reproducing the native N-terminal sequence of the target protein. To analyze the impact of these linker sequences on export yields, the clones with both the cleavage sites were constructed first with a mouse variant of TNF-α. Given that mouse and human TNF-α are fairly similar proteins, sharing a 79% homology in their amino acid sequences, we assumed that the impact of different linkers on extracellular protein expression would be similar for both proteins. Expression studies were conducted using the previously optimized conditions, and samples were run on SDS-PAGE.

**FIGURE 7:**
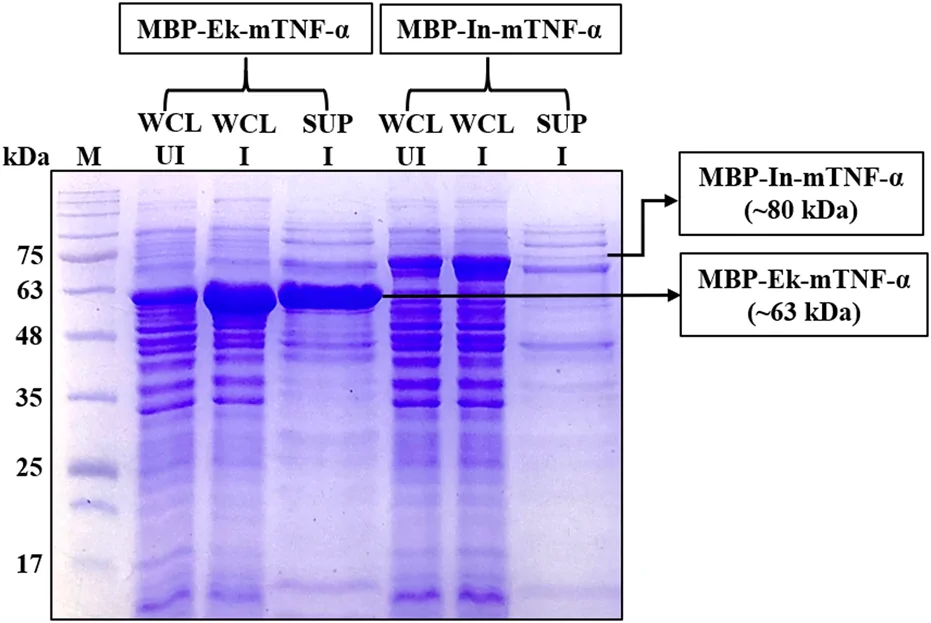
SDS-PAGE showing 40 h post-induction co-expression profile of MBP-Ek-mTNF-α and MBP-In-mTNF- α with p^PROLAR.A^SecAB in DKO strain. High export was observed with the enterokinase cleavage site (Ek), whereas intein (In) showed minimal export. M, Molecular weight marker; UI, Uninduced; I, Induced; WCL, 10 µL of 10 OD_600_ equivalent whole cell lysate sample; SUP, 20 µL of culture supernatant.

We observed that both the cytoplasmic and supernatant fractions of MBP-mTNF-α were significantly higher when the enterokinase cleavage site was used as the linker sequence (Figure 7). Interestingly, the yield of mTNF-α in culture supernatant was slightly higher than that of hTNF-α. In the case of intein, the intracellular accumulation of the fusion protein was comparatively low, and export of the target fusion protein was not observed. The protein-partitioning of MBP-In-mTNF-α showed soluble expression of the fusion protein, and the soluble fraction was purified using amylose affinity resins (data not shown). The self-cleavage reaction was set up at different temperatures (25 °C and 37 °C) and pH (6 and 7), but we did not see cleavage under any of the conditions tested (Supplementary Figure S2). Since intein, as a cleavage site, completely blocked export and did not even show any cleavage of the fusion protein, the enterokinase cleavage site was chosen to be inserted in MBP-hTNF-α for further downstream processing. Clearly, the small size of the linker was crucial in preventing any adverse impact on export efficiency.

### 3.8 Expression and purification of MBP-Ek-hTNF-α

**FIGURE 8:**
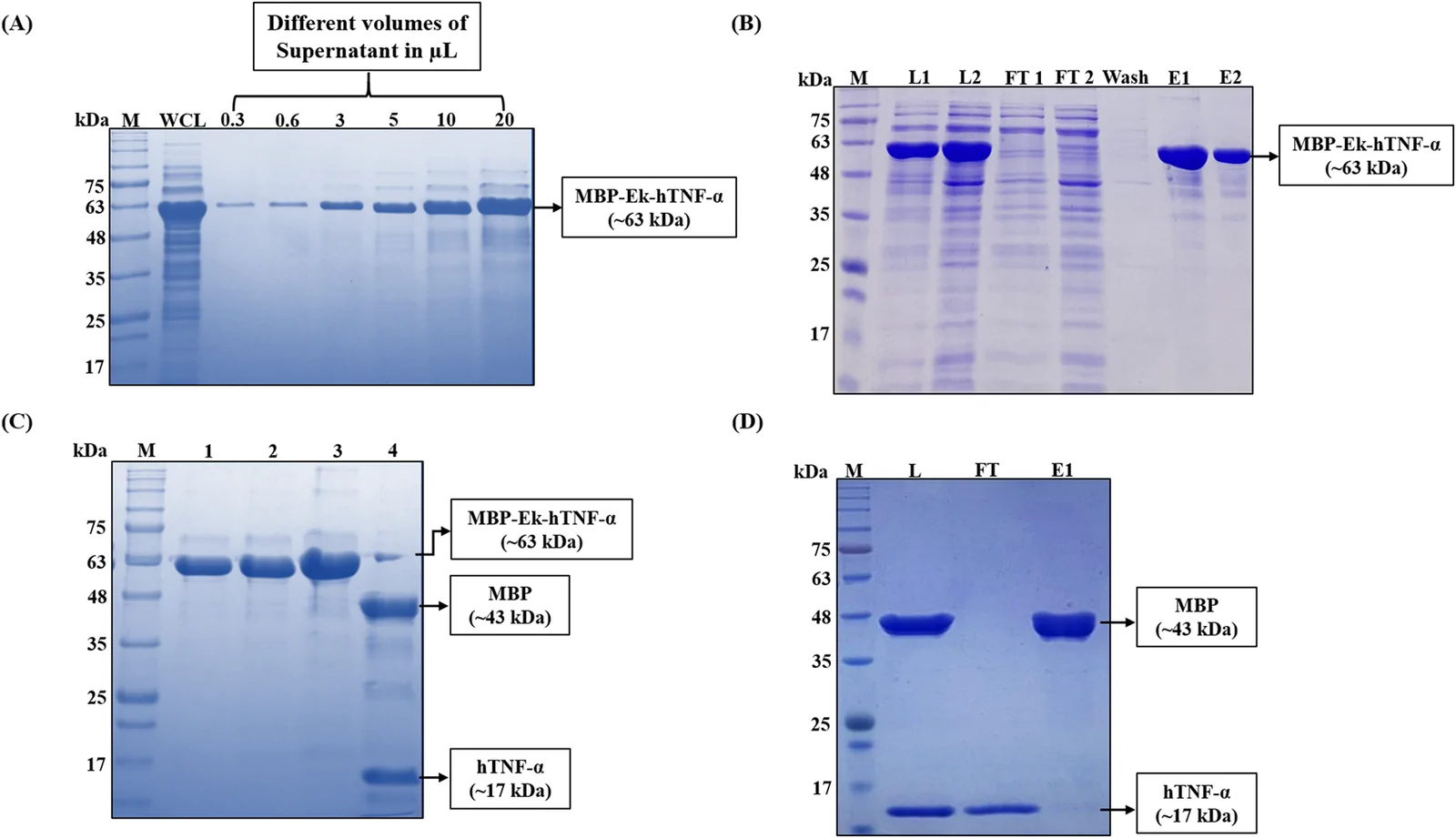
**(A)** SDS-PAGE showing 40 h post-induction co-expression profile of MBP-Ek-hTNF-α and p^PROLAR.A^SecAB in DKO strain. A clear band of MBP-Ek-hTNF-α was observed in as low as 0.3 µL extracellular supernatant sample, representing very-high extracellular yields. M, Molecular weight marker; WCL, 20 µL of 10 OD_600_ equivalent whole cell lysate sample; SUP, Different volumes of extracellular supernatant fraction **(B)** SDS-PAGE showing affinity purification of MBP-Ek-hTNF- α using dextrin sepharose high-performance resins from Cytiva. The fusion protein appeared as ∼95% pure. M, Molecular weight marker; L1, Supernatant fraction; L2, PBS wash + supernatant as final load; FT1 and FT2, flow-through collected at different time points; Wash, wash with equilibration buffer; E1 and E2, elution fractions collected at different time points **(C)** SDS-PAGE showing cleavage of MBP-Ek-hTNF-α using bovine enterokinase (bEkL). M, Molecular weight marker; 1,2,3, Different volumes of control (MBP-Ek-hTNF-α) 4, Digestion of MBP-Ek-hTNF-α using bEkL **(D)** SDS-PAGE showing purification of hTNF-α using DEAE Sepharose fast-flow resins from Cytiva. Native hTNF-α was obtained as >98% pure. M, Molecular weight marker; L, Digested MBP-Ek-hTNF-α; FT, flow-through (hTNF-α); E1, elution fractions collected (MBP).

As in the case of mTNF-α, the enterokinase cleavage site was inserted between the fusion tag and target protein hTNF-α via PCR (Supplementary Figures S3A–C). The positive clones were confirmed by restriction digestion (Supplementary Figure S3D) and sequencing. The modified clone was co-expressed with p^ProLar.A^SecAB at optimized conditions, and samples were run on SDS-PAGE. The pH of the culture during pulse addition was ∼8.2, and at the time of harvesting was ∼8.5, which facilitated export. The intracellular fraction (which was ∼30% of total cell protein) and the extracellular fraction were found to be similar as that of previous results when we had used a flexible linker without any cleavage site. Different volumes of supernatant were loaded, and the fusion protein could be clearly observed in as low as 0.3 µL of the extracellular supernatant (Figure 8A). The final OD_600_ of the culture was observed as 30. Therefore, 33.3 µL of the culture was pelleted (equivalent to 1 mL of 1 OD_600_ culture). This pellet was resuspended and lysed in 100 µL 1X SDS-PAGE loading dye (making it equivalent to 10 OD_600_ culture). This represents a 3-fold dilution of the original culture. An equivalent of 6.7 µL of this culture pellet was loaded (since we loaded 20 µL of a 10 OD_600_ equivalent culture) and compared with different volumes of the supernatant to determine the relative ratio of the protein in the intracellular vs. extracellular fraction, and we estimated that ∼40% of the total fusion protein got exported from the cytoplasm to the culture medium.

It has been previously shown that sometimes the MBP fusion-tagged protein appears soluble even when the target protein is misfolded and insoluble (Nominé et al., 2001; Hewitt et al., 2011). To confirm that our production strategy led to a soluble and bioactive target protein, we decided to purify the protein and test its biological activity in L929 cells.

For this, a 2-step purification was done using the BIORAD purification system to obtain native and pure hTNF-α. The extracellular supernatant obtained after centrifugation was loaded onto a column containing amylose affinity resins. The MBP-Ek-hTNF-α was eluted using 50 mM maltose. The protein was found to be ∼95% pure (Figure 8B), and the densitometry analysis showed >95% recovery of the fusion protein in the elution fraction by comparison with the original supernatant. The concentration of this purified protein was calculated after volume correction by Bicinchoninic Acid (BCA) assay and found to be 1.3 g/L. We are therefore reporting the original concentration of the fusion protein also as ∼1.3 g/L, assuming 100% recovery, which is a conservative estimate given the fact that there would have been some minor losses in the previous 2 purification steps. Elution samples were utilized to set up an overnight digestion reaction (Figure 8C) with in-house produced bovine enterokinase (bEkL) at 4 °C (Akanksha et al., 2025).

Following digestion and buffer exchange to 20 mM Tris pH 7.5 + 50 mM NaCl, the sample was loaded on a DEAE column. The target protein (hTNF-α) was obtained in the flow-through fraction, whereas MBP was eluted later at 500 mM NaCl concentration. The overall recovery of native hTNF-α was calculated as 80% by densitometry of the SDS-PAGE samples, and thus the final yields were ∼380 mg/L of pure hTNF-α as also confirmed by a Bicinchoninic Acid (BCA) assay.

### 3.9 Biological activity assay of purified recombinant hTNF-α

The bioactivity of the purified recombinant hTNF-α was evaluated by cytotoxicity assay of L929 cells. L929 cells were cultured in Minimum Essential Medium, fetal bovine serum, and penicillin-streptomycin for cytotoxicity measurement. Each well was filled with 100 µL of culture medium with 2 × 10^5^ cells/mL, and incubated overnight in a 37 °C/5% CO_2_. After 24 h of incubation, the culture medium was replaced with 100 μL of culture medium containing 1 μg/mL actinomycin D and 0.1–1,000 pg/mL of rhTNF-α, followed by incubation for 24 h. 10 μL of CCK-8 solution was added for 2 h, and the optical density was determined at 450 nm and 620 nm in comparison to the control.

**FIGURE 9:**
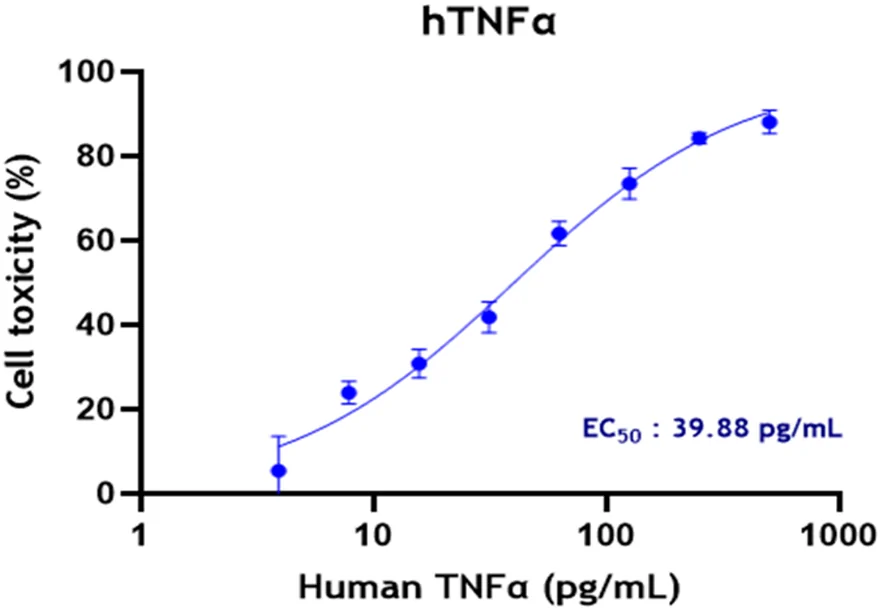
L929 cytotoxicity assay of purified hTNF-α. The purified recombinant protein was found to be active.

The L929 cell assay was carried out to analyze the bioactivity of purified rhTNF-α. The dose–response curve ranging from 0.1 pg/mL to 1,000 pg/mL showed an EC50-value of 39.88 ± 5.5 pg/mL (Figure 9). The EC50 value obtained was well within the range of the standard EC50 values of 25–100 pg/mL as reported in literature, indicating that the purified rhTNF-α in our experiment was biologically active, with an activity of approximately 2.5 × 10^7^ U/mg.

## 4 Discussion

Two synergistic breakthroughs allowed us to design this technology for efficient export of recombinant proteins. The first was the use of a DKO (Double knockout) strain, which blocked the cellular stress response and hence sustained protein expression for long periods. The second was the serendipitous discovery that MBP-tag with a small linker (enterokinase cleavage site in this case) not only solubilized the target protein but also facilitated export. It is interesting to speculate whether the DKO also had a role in enhancing export by somehow impacting the outer membrane permeability. However, we notice that the MBP alone did not get exported in the DKO host, while the MBP-hTNF-α got exported, albeit at low levels, even with the *E. coli* BL21 (DE3) host (Supplementary Figure S4). Both these observations underscore the primary role of protein structure in export, with the DKO essentially being a necessary but not a sufficient condition for the buildup of high extracellular titers. This by itself can be considered a positive feature of this strategy since it guarantees a highly specific export with little or no host cell protein (HCP) contamination in the supernatant. This is unlike the previous strategy by other researchers (Shin and Chen, 2008; Chen et al., 2014; Yang et al., 2018; Ding et al., 2019; Hu et al., 2019), where enhancing outer membrane permeability improves extracellular titers at the expense of higher levels of contamination by HCPs. Finally, the true potential of the DKO host can now be realized because the sustained buildup of the target protein is no longer limited by the space constraints imposed by the intracellular cell volume. We can also safely postulate that the role of SecA/SecB and the linker peptide is limited to enhancing translocation, and even the native leader peptide of malE is a more efficient translocator of MBP compared to pelB when secretion to the periplasmic space is concerned. All the observations imply that sufficient export is a two-step process with an initial buildup in the periplasm being the forerunner of subsequent transport across the outer membrane. This second step is highly specific to the nature of the protein and, interestingly, pH-dependent since export took place only when pH was >8.0. In order to convert our export strategy into a more generic technology for extracellular protein production, we did some preliminary studies on a basket of cytokines tagged with MBP. Interestingly, about half the cytokines studied were efficiently exported while the other half were not. This was in spite of the fact that they were produced in soluble form in the cytoplasm. We speculate that the presence of a lesser number of disulfide bonds, which makes the target protein less rigid, and the hydropathy score of the protein are important determinants of export.

## 5 Conclusion

The selection of a knockout BW25113 Δ(*elaA + cysW*), which sustained protein expression, allowed us to design genetic and bioprocess strategies that facilitate the slow and specific export of the protein. Thus, a combination of vector design, which includes the promoter, the N-terminal tag, and the linker sequence along with co-expression of pSecAB formed part of the genetic strategies which enhanced secretion. The bioprocess strategy of using rich media and pulsing of nutrients to maintain the cellular health for long periods combined with a lower temperature to prevent misfolding helped continue the expression for long periods thus allowing a very high buildup of a very clean recombinant protein product in the supernatant. Thus, while the intracellular protein concentration could be estimated at ∼1.8 g/L, a fairly large fraction of ∼1.3 g/L also got exported to the supernatant. We can conclude that the efficient export of proteins also frees up the intracellular space, allowing for a longer period of unhindered protein production. Finally, it is interesting to note that the total amount of protein produced if we add both intracellular and extracellular fraction would represent >50% of the total cellular protein, a value which would be impossible to attain for soluble proteins, unless a significant fraction is continuously exported outside the cell.

We demonstrate here a strategy of high-level and extremely target-specific export of recombinant proteins in *E. coli,* which, because of its obvious advantage, may help lead a technological shift in protein production methods. However, a comprehensive analysis of the role of the protein structure in facilitating export is required before this methodology can be applied to a larger basket of proteins.

## Data Availability

The original contributions presented in the study are included in the article/Supplementary Material, further inquiries can be directed to the corresponding authors.
