# Supplementary material for: Designing an optimized strategy for extracellular expression of recombinant human TNF-α in Escherichia coli
Source: Front Bioeng Biotechnol. 2026 Aug 28;14:1897529. doi: 10.3389/fbioe.2026.1897529 (PMC13562010; doi:10.3389/fbioe.2026.1897529)
Supplement: Supplementary file 1 [file Supplementaryfile1.docx]

***Supplementary Material***

**Supplementary Figures**

**Supplementary Figure S1.** Visualization of MBP-hTNF-α export in the extracellular medium with *malE* and *pelB* as signal peptide sequences. Expression studies were conducted till 40 hours post-induction. *malE,* as a signal peptide, showed better export of MBP-hTNF-α as compared to *pelB*. **Lane M**-Molecular weight marker; **Lane WCL**- 10 µL of 10 OD_600_ equivalent whole cell lysate sample**; Lane SUP-** 20 µL of culture supernatant**.** Legends: **UI**- Culture incubated at 25 ℃ without IPTG induction; **I**- Culture incubated at 25 ℃ with IPTG induction.

**Supplementary Figure S2.** Visualization of intein cleavage using soluble and purified MBP-In-mTNF-α at different pH (including pH 6 & 7) and temperature (including 25 ℃ & 37 ℃) combinations to obtain native mTNF-α. Self-cleavage of the intein was not observed under any condition tested. **Lane M**-Molecular weight marker; **Lane CTRL**- Uncut control of MBP-In-mTNF-α.

**(A)	(B)**

**(C)	(D)**

**Supplementary Figure S3.** **(A) Agarose gel showing gradient PCR at different temperatures.** **Lane L-** 1 Kb DNA Ladder; **Lane 1-** 56 ℃; **Lane 2-** 57.9 ℃; **Lane 3-** 59.2 ℃; **Lane** 4- 61 ℃; **Lane 5-** 62.5 ℃ **(B) Agarose gel showing EcoRⅠ and HindⅢ digested pMAL-p2X vector.** **Lane L-** 1 Kb DNA Ladder; **Lane 1&2-** EcoRⅠ and HindⅢ digested pMAL-p2X vector; **Lane 3-** Uncut pMAL-p2X vector **(C) Agarose gel showing colony PCR to identify a positive clone of MBP-Ek-hTNF-α.** **Lane L-** 1 Kb DNA Ladder; **Lane 1 to Lane 5-** Various colonies amplified at 56 ℃ to screen positive clone; **Lane 6-** Negative control; **Lane 7-** Positive control **(D) Agarose gel showing clone confirmation of MBP-Ek-hTNF-α by double digestion using EcoRⅠ and HindⅢ restriction enzymes. Lane L-** 1 Kb DNA Ladder; **Lane 1 & 2-** Positive colonies digested with EcoRⅠ and HindⅢ. Both colonies showed insert (489 bp) and vector (~6.7 kb) at their respective sizes following digestion**.**

**Supplementary Figure S4.** SDS-PAGE showing the expression profile of MBP-hTNF-α in BL21 (DE3), BW25113, and DKO *E. coli* strains. Expression studies were conducted till post-induction^.^40 hours. DKO showed the highest extracellular export of MBP-hTNF-α as compared to the other two strains. **Lane M-** Molecular weight marker; **Lane UI-** Uninduced; **Lane I-** Induced; **Lane WCL-** 10 µL of 10 OD_600_ equivalent whole cell lysate sample; **Lane SUP-** 5 µL of culture supernatant.

**Supplementary data**

**Detailed Bioassay Protocol**

# Complete medium composition –

MEM, 10% FBS, 1% PS

# Assay medium composition –

MEM, 10% FBS, 1% PS, 1μg/ mL Actinomycin D

# Step 1: Cell Seeding:

1. All the manipulations with growing cells were performed in a tissue-culture flow hood, and cells were grown until 75-85 % confluent in complete culture medium.
2. 1.5 mL of 1X Trypsin-EDTA was added to the T- Flask and left in the incubator 2-5

min until the cell layer was dispersed.

3. The flask was removed from the incubator, and 3mL complete media was added. Gently pipetted up and down 8-10 times to get a single-cell suspension.
4. Cells were added to a 15 mL Falcon and centrifuged at 10000 rpm for 05 mins at room temperature.
5. Cells were then washed 2times with assay medium, and the pellet was resuspended in 5 mL of the assay medium.
6. 15 µL of cells was added to 15 µL of Trypan blue, and counted using a haemocytometer, and the cell suspension was adjusted to 2 * 10^5 cells/mL.
7. 100 µL of the cell suspension (2 * 10^3 cells) was added to each well of the 96-well flat-bottomed tissue culture plate, leaving all the outside wells empty. The outer wells were filled with 150 μL of PBS to prevent evaporative loss.
8. The cells were incubated in a humidified 37°C incubator with 5% CO2 for 24 hours.

# Step 2: Sample Preparation and Cell Treatment: Reconstitution buffer: PBS + 0.1% BSA

Dose required (pg/mL or ng/mL): 0, 3.90, 7.81, 15.62, 31.25, 62.5, 125, 250, 500 pg/mL

Dose prepared (pg/mL or ng/mL): 0, 3.90, 7.81, 15.62, 31.25, 62.5, 125, 250, 500 pg/mL

1. 100 μL of each dilution was added in triplicate to the plate. The following control wells were also setup:
   - Blank control wells: Contain only culture medium and CCK-8 reagent (no cells).
   - Untreated control wells: Contain cells and culture medium without any test compounds.
   - Positive control wells: Include appropriate controls based on the experiment.
2. After 24 hours of incubation at 37°C in humidified air with 5% CO2, 10 μL CCK-8 was added to the wells. After 2 hours, the plates were read in a microplate reader at 450 nm & 620 nm, and the data was evaluated using the GraphPad Prism software. The EC50 value was calculated using the 4-Parameter Logistic (4PL) regression in the nonlinear curve-fit model.
